# Treating Schizophrenia: Open Conversations and Stronger Relationships Through Psychoeducation and Shared Decision-Making

**DOI:** 10.3389/fpsyt.2020.00761

**Published:** 2020-08-13

**Authors:** Armida Mucci, Wolfram Kawohl, Cristiana Maria, Annette Wooller

**Affiliations:** ^1^Department of Psychiatry, University of Campania Luigi Vanvitelli, Naples, Italy; ^2^Department of Psychiatry and Psychotherapy, PDAG, Brugg, University of Zurich, Zurich, Switzerland; ^3^Communications EMEA, Janssen Pharmaceutica NV, Beerse, Belgium; ^4^Medical Affairs EMEA, Janssen Pharmaceuticals, High Wycombe, United Kingdom

**Keywords:** psychoeducation, schizophrenia, carers, people living with schizophrenia, shared decision-making

## Abstract

Integrated pharmacological and psychosocial treatments, such as psychoeducation (PE) and shared decision-making (SDM), have been shown to significantly improve outcomes for people living with schizophrenia (PLWS). Underpinning the success of these interventions is a strong therapeutic relationship between PLWS, their carers, and their healthcare team. While many recognize the value of this relationship, implementation of the interventions necessary to facilitate its construction remain low. In this article, we identify the barriers to developing productive therapeutic relationships and explain how PE and SDM, taking into account cultural difference, can improve adherence to treatment, strengthen therapeutic relationships, and ultimately equip patients to achieve better functional outcomes.

## Introduction to the Biopsychosocial Model in Schizophrenia and the Importance of Psychoeducation

Schizophrenia is a complex disorder that is impacted by biological, social and psychological factors. Recent years have seen a shift towards how psychosocial factors, such as isolation, victimization, and substance abuse, can influence outcomes (1). The biopsychosocial model of schizophrenia considers the cause and course of schizophrenia as equally related to biological vulnerability interacting with social and psychological factors, e.g., isolation and low self-esteem, with recommended psychological interventions based on individual requirement (2, 3).

We believe that optimal outcomes for people living with schizophrenia (PLWS) arise when healthcare professionals (HCPs) consider both pharmacological and psychological interventions. This enables them to individualize each case and implement treatment plans tailored to their history, family, and cultural background. In clinical practice, pharmacological therapies are well understood and widely implemented however, psychosocial treatments are not. Therefore, we need to embed psychosocial treatment into standard care for PLWS and improve the therapeutic alliance between PLWS, their carers, and their healthcare team (4).

This article aims to identify barriers to the achievement of strong therapeutic relationships and highlight the benefits of consistently implementing psychosocial treatments such as psychoeducation (PE), and shared decision-making (SDM).

PE involves providing accurate, relevant and up-to-date information to PLWS as well as to their carers and family (5). PE focuses on improving insight and giving practical support on managing the condition. SDM promotes collaboration between patients and clinicians, where information is shared and patients are supported to express and achieve informed preferences about their treatment (6).

This review article uses the biopsychosocial model to discuss the personalization of care for PLWS, focusing on the importance of knowledge and expertise in pharmacological and psychosocial aspects of schizophrenia and the importance of implementing an individualized approach that is sensitive to patients’ history and cultural backgrounds. We believe that embedding PE and SDM into the interactions between PLWS and their healthcare teams will help improve therapeutic relationships and subsequently overall outcomes.

## Establishing and Addressing a Need for Open Conversations and Strong Relationships

Clinicians should aim to build a strong therapeutic alliance between themselves, the patient and their primary carer(s). An alliance should be non-hierarchical, acknowledging the respective expertise of the individuals, with all members given respect and made to feel like partners with a common goal (7).

To construct a strong therapeutic alliance, open conversations, trust and respect play a fundamental role, with the ultimate goal being to establish a partnership to optimize patient outcomes. Strong therapeutic alliances have been shown to correlate with positive patient outcomes, notably symptom severity, hospitalizations, rates of drop-out from psychosocial treatment, and adherence to medication (8). This article discusses how various barriers specific to individual patients, such as social and cultural factors, influence the formation of strong therapeutic alliances.

## Recognizing the Role of Carers

It is important to recognize all stakeholders involved in the care of PLWS; the role carers play should not be undervalued. Carers can relay important information about how the PLWS is coping, how the person is daily, and whether any change in wellness or behavior has occurred. It is important to note that carers may or may not be family members.

## Barriers to Strong Relationships

There are numerous patient, carer, and clinician barriers that can impair the formation of relationships; these vary between cases and vary from misinterpretation of patient insight to appreciation of cultural background.

## Patient Barriers to Strong Relationships

### Insight

“Insight” describes the ability of PLWS to recognize that they have an illness and their ability to understand how their experiences relate to the illness (9). In our experience, insight exists on a continuum; patients often recognize some aspects of their symptomatology, but not others. For example, formal thought disorder and hallucinations are better recognized as pathological, while delusions are less so (10). Poor insight is a known feature of schizophrenia and has been linked to poorer perceived therapeutic alliance (8, 11, 12).

Impaired insight can hinder relationships, not least due to the negative effects this has on social interaction and perception of the actions of others (13). If a person does not believe that they have an illness, then any attempt to address it will be futile. Crucially, this can lead the person to distrust the help provided by carers and professionals. It is important to gauge the baseline level of insight to provide suitable individualized PE (14, 15). Although appreciation of insight prior to providing PE is beneficial, providing quality PE to all PLWS regardless of insight is beneficial. In PLWS, PE is one of the most consistently effective treatment modalities, with relapse rate reduction at 50%–60% over treatment as usual (5).

### Stigma

Stigma describes a negative perception or judgement towards a person, which can lead to alienation and discrimination. For PLWS, stigmatization (from others and self) causes embarrassment, insecurity and stress. Stigma can lead to communication problems, including a reluctance to talk openly about their illness. Stigma changes how subjects perceive themselves—leading to feelings of incompetence and low self-esteem. Stigma correlates with poorer real-life functioning as measured by maintenance of close and extended social circles, productivity at work and independent living (16–18). PLWS often use social withdrawal as a mechanism for coping with stigma, which can have a negative impact on relationships and impede the desire to discuss and share during consultations.

Cultural context can affect family burden, stigma and the progression of schizophrenia. A predictor of poor course in schizophrenia is highly expressed emotion (EE), which varies widely across ethnic groups (19). Furthermore, differing levels of acceptance of schizophrenia and criticism towards PLWS exist between different cultural groups, e.g., Anglo-Americans tended to blame their affected relatives more than Latino Americans (20). Additionally, gender inequality is strongly affected by cultural factors and in certain regions, female PLWS may be devalued and submitted to excessive emotional stress, violence, isolation and denial of access to care (21). Cultural and gender-informed therapy should be offered to PLWS and their families. Therapists should be aware that PE, other psychological therapies and the therapeutic alliance may be severely impacted by these factors. For example, a delay in seeking help may be associated with high levels of stigma in some cultures, e.g., Afro-Caribbean immigrants in the UK delayed access to care for PLWS, with an increase in the duration of untreated psychosis associated with a subsequent worsening of outcome (22).

## Healthcare System/Psychiatrist Barriers to Strong Relationships

### Poor Provision of Information

Hesitancy from HCPs in sharing information with PLWS and their carers can have a negative impact on outcomes. In our experience, hesitancy occurs due to several factors including poor insight, insufficient training on PE technique, and the physician’s fear of offending and disrupting the relationship with the patient. Sometimes even core pieces of information, such as the diagnosis of schizophrenia, are initially omitted, with vague terminology, e.g., “a disorder of the brain” used, which may be confusing, or even inaccurate (23, 24). Avoiding use of “schizophrenia” may prevent PLWS from seeking the treatment and support required and delay the opportunity to build a strong relationship (24).

Most people with severe mental illness have a high desire for information. This information is associated with variables, such as therapeutic relationship and symptom severity, which are amenable to change during treatment (25).

Poor communication of information extends to conversations about treatments. Some clinicians fear that informing PLWS about options like long-acting therapies too early may be viewed as suggesting that PLWS are untrustworthy, their condition is more severe, or that clinicians do not want to see them (26–28). In our experience, open discussion about the need for pharmacological treatment is fundamental and should always happen as early as possible. Discussions around drug options can be influenced by poor insight of PLWS, especially during the acute phase, and PLWS with persecutory ideas involving carers and staff, with no illness insight, may not be able to fully consent to treatments.

A poor understanding of cultural context may inhibit a HCP’s ability to form an open, honest and transparent relationship with their patient. However, the integration of culturally-based treatment approaches with existing PE interventions are now available, or are being developed for PLWS from ethnic minority groups, including Hispanic, Latino, African, and Caribbean communities (29, 30). This is in accordance with the NICE Schizophrenia Guidelines, which state that “services should also ensure that all clinicians are skilled in working with people from diverse linguistic and ethnic backgrounds, and have a process by which they can assess cumulative inequalities through their routine clinical practice” (31).

Despite strong guideline recommendations of education for PLWS and carers, provision of PE remain low, with a minority of clinicians and nurses receiving relevant training (12, 31–34).

### Lack of Patient-Centered Outcomes

PLWS generally value different outcomes to those considered by HCP’s and this can create barriers in forming therapeutic relationships. While HCPs value outcomes are usually symptom relief or relapse prevention, PLWS may desire outcomes such as acceptance by their family or peers, or a return to school or work. These valued outcomes are not addressed in certain therapeutic settings, yet are invaluable in building strong therapeutic relationships. Discussing and setting patient-centered outcomes may build hope and help trust in the therapeutic relationship (35).

### Outdated Attitudes

Sometimes, important aspects of personal life, like sexual activity and illicit drug use, are avoided in clinical discussions, which can create distrust. Avoiding discussions about sexual activity might suggest to PLWS that sexual disturbances due to treatment are being overlooked. Interestingly, patients are often willing to talk about these aspects when prompted (36).

Cultural factors directly impact relationships. Individual perceptions of schizophrenia and cultural context may affect the likelihood of the PLWS to seek help or be transparent about their experiences and their views of the role of their loved-ones in helping to fulfil the carer role (37).

PLWS and clinicians sometimes differ when defining treatment goals and outcomes. Patients often prioritize life goals and real-world problems, whereas clinicians usually place emphasis on symptoms, such as hallucinations (4). Patient-related outcomes should be valued and complement other outcome measures (38). A patient’s job and housing situation should also be considered.

## Carer Barriers to Strong Relationships

Carers’ behaviors and attitudes can have significant effects on social functioning and treatment success. Often, stigma and a lack of understanding or acceptance from families may result in poor support for PLWS, which can impact their condition.

The influence of cultural context also extends to the carer. In some cultures, it may be considered humiliating and/or offensive to involve a relative younger than the patient, or a female peer in therapy discussions (37, 39).

EE can refer to positive or negative communication strategies used by a carer towards PLWS. The construct of EE includes five components: critical comments, emotional over-involvement, hostility, warmth, and positive remarks (40). The first three components are associated with poor outcomes while warmth and positive remarks are protective factors (41). Higher levels of negative EE correlate with poorer compliance and predicts poorer social functioning for PLWS (41, 42). The presence of these aspects is also influenced by the cultural context, for example, EE rates are higher in Western cultures (43).

## Socioeconomic Barriers

Clinicians should be mindful of the socioeconomic and organizational barriers to maximizing their contact time with PLWS. These are typically beyond the control of individual clinicians, but awareness may help guide practice. In our experience, barriers that can reduce contact time include frequent changing of facilities and staff, high caseloads for clinicians, unstable living situations for PLWS, or unavailability of accessible transport to get to appointments. A broader availability of well-organized outpatient and outreach mobile services can improve early detection and the quality of the first therapeutic relationship that is crucial for treatment adherence. Studies have shown that early recognition and treatment improves outcomes for PLWS and reduces individual and societal costs (12, 44).

## Addressing the Barriers to Achieve Stronger Relationships

### Greater Attention and Support for Carers

Studies have shown that having a family member or carer who provides informal support is associated with better treatment compliance (45, 46). Keeping carers closely involved in the treatment plan helps to open up other channels of communication for the clinician and PLWS.

It is important to make sure the patient-clinician-carer dynamic is positive; if this deteriorates, revisiting the roles of carers, when they should come to appointments, and whether changes are needed can help restore balance.

### PE for Carers and PLWS

#### Benefits of PE for Carers and PLWS

PE is a useful tool for improving relationships within the care alliance. The clinical benefits of PE have been demonstrated in numerous studies and include positive impacts on clinical measures, e.g., relapse rates, relapse severity, and adherence (14, 47, 48). Regarding promoting strong therapeutic relationships, PE has been shown to improve patient insight, reduce self-stigma and improve social functioning (14, 49, 50). However, while improving insight is a key goal of PE, even patients with high levels of insight may still benefit (5).

PE also improves outcomes for the carers and family members of PLWS. Studies exploring family therapy approaches have shown that PE reduces carer stress and hostility towards other family members, while increasing warmth and reducing the perception of burden (51–53).

#### What Should PE Cover?

PE will inevitably include schizophrenia-specific information, such as how to recognize symptoms, the impact of the illness on real-life functioning and the importance of treatment for optimal outcomes. Clear and essential information should be provided, and basics of the vulnerability-stress model should be addressed to adequately present the need for treatment. Promoting healthy lifestyles, identifying causes of stress, and problem-solving and communication skills are equally important domains of PE (5).

A large part of the learning from a PE intervention will likely take place outside of the clinician consultation; the clinician plays a critical role in directing PLWS and their carers to reliable and easily-understandable sources of information, such as e-mental health interventions (54). Guidelines recommend that educational interventions, including those surrounding treatment options, are introduced as early as possible (31, 32).

Different PE approaches should be considered for PLWS and their carers. PLWS may benefit more from an approach that enhances problem-solving skills and promotes the identification and achievement of life goals. For carers, greater importance may be placed on the symptoms of schizophrenia, with a focus on promoting acceptance (51). Healthcare providers should consider how better access to PE can be provided in general to PLWS and carers (34). Importantly, all information should be accurate, fair and balanced, and, when possible, not influenced by preconceived attitudes and opinions of the clinician.

### Shared Decision-Making and Patient Partnership

While clinicians are experts of the illness, PLWS, and carers are experts of themselves or experts by experience, and the contribution that each can bring to the decision-making process should be recognized and considered. Unfortunately, although there is strong support for SDM among PLWS and psychiatrists, evidence suggests that this does not routinely take place, often due to various different factors (55–57). Commonly-identified barriers to SDM include poor insight and lack of decisional capacity from the patient, societal and cultural expectations about psychiatric disorders, beliefs surrounding the effects of treatments, and financial and timing pressures which can limit options (35, 58).

There are a number of factors that can be incorporated into consultations to help facilitate SDM. These include honesty, trust, respect and politeness from all parties (7). Honesty is required from the clinician (e.g., regarding treatment options and potential side effects), but also from PLWS and carers (e.g., reporting symptoms or adherence) (7). In our view, a warm attitude from the treatment team, a manifest interest for the person and his/her opinions, and empathetic behavior towards distress caused by treatment or symptoms can help establish a correct and trustful therapeutic atmosphere even for PLWS with poor insight.

Open discussion of treatment options should happen as early as the patient is ready, with evidence suggesting that early introduction of both pharmacological and psychosocial treatments for schizophrenia can positively impact overall treatment success (27, 28, 32, 59), and positively impact SDM (32, 59).

In our view, following pharmacological treatment guidelines is the gold standard of treatment in schizophrenia in order to prevent hospitalization, relapse, and to promote community independent living which is imperative for patient recovery. When considering the recommended treatments, discussions should always include the pros and cons of different options, and the treating team should routinely consider whether the current treatment is optimal or whether there might be better needs-oriented options. **Table 1** contains a list of questions, parameters and solutions that can be implemented to help uncover and address patients’ needs and attitudes towards their current treatment. Everyone should be open to the idea of revisiting treatment discussions to find the best option for the patient. Therefore, we believe that the shared view of schizophrenia and its treatment that has to be reached in the therapeutic relationship should address biological vulnerability and the positive role antipsychotics have in treatment.

**Table 1:** Example questions, parameters, and solutions to consider when assessing the success of a patient’s treatment.

**Questions for the patient regarding their wellbeing and treatment**	**Parameters to consider for reviewing the treatment plan**	**Side effect/emergent condition to address**	**Possible solution**
How have you been feeling since your last appointment? What activities have you been doing since we last met? Do you have any questions you would like to ask?	N/A	General adherence	Encourage patient empowerment
Have you experienced any problems since your last visit? (e.g., fever, rash, fatigue, etc.)	Blood count, electrocardiogram	Common or rare drug side effects	Revise therapy taking into consideration drug interactions, intolerance, and common or rare side effects
Have you noticed an increase in appetite or weight? Has this been a problem for you?	Body weight, waist circumference, body mass index (BMI), glycaemia, serum lipid profile	Metabolic syndrome	Prescribe an antipsychotic with less of an impact on metabolism/weight
Have you noticed any irregularity in your menstrual cycle (females only), any discharge of milk from your breast or breast enlargement (either sex), a reduction of sexual drive, or other dysfunctions related to your sexual life?	Prolactin levels	Hyperprolactin-emia	Prescribe a different antipsychotic or prescribe an additional agent to lower prolactin
Have you felt demotivated or indifferent more than usual? Has anyone noticed that you are less active or expressive than usual? Do you feel sad most of the time?	N/A	Secondary negative symptoms due to extrapyramidal side effects or depression	Switch to a second generation antipsychotic or treat depression separately
Do you feel clumsy or slowed-down in your movements or unbalanced when standing? Do you notice a tremor when resting?	Muscle stiffness	Drug-induced parkinsonism	Switch to a second generation antipsychotic with low potential to induce parkinsonism; add an anticholinergic drug
Have you been able to take your medications regularly or did you need to be prompted? Would you prefer another schedule? Would you feel more comfortable if we simplified your schedule?	N/A	Adherence	If preferred by PLWS or deemed important by the doctor, a switch to a long-acting injectable antipsychotic should be considered

It is important to involve PLWS in treatment choices; you should openly discuss their attitudes towards drug or psychological treatment and allow their preferences to be stated without judgmental comments. Within this context, it is useful to frame pharmacological interventions as a way to reduce the distress related to symptoms and protect the patient against the stress-related development of these symptoms. A schizophrenia diagnosis should be discussed at the earliest opportunity, ideally when the team are confident of its veracity; if there is insufficient evidence, a provisional diagnosis should be communicated, explaining that further information is required for a definitive diagnosis.

Patients can be encouraged to take a more active role in their treatment by asking open-ended questions, taking time to consider their viewpoints, verifying their ability to understand information and instilling them with confidence about their involvement. PE and a strong patient partnership will often come hand in hand; more proactive and engaged patients are typically more informed regarding their treatment (7).

### Formal PE Training for Clinicians

Psychiatrists should consider upskilling themselves and be aware of the benefits this could have for patient outcomes. Studies have shown that settings-based training programs can improve therapeutic relationships (60, 61).

Psychiatrists should be encouraged to access materials and courses that can further help them apply PE. Those who have more experience with PE should consider furthering their reach by advocating for widely available PE training within their countries, working closely with scientific professional societies to ensure that they produce more accessible opportunities for training, and promote awareness of the importance of PE within their healthcare communities.

## Discussion

There are several key components that are necessary to create a strong treatment plan for PLWS including; a strong therapeutic alliance, SDM, and PE. Each of these components build on the benefits of the others; for example, patients that are more educated regarding their condition are able to build stronger therapeutic relationships and engage in SDM. Likewise, patients that are more active decision-makers are more likely to seek out education and adhere to their treatment plans.

We believe that the role carers play in the treatment of PLWS requires wider recognition. Carers can have positive and negative influences on outcomes for PLWS, which is why choosing the right person to support PLWS is imperative. As clinicians, we can help guide conversations about who is the patient’s primary carer and how much they are involved, while considering any socio-economic and cultural factors, but decisions around this should ultimately belong to the patient. Having a supportive, reliable, and engaged carer should be a key ambition for all those involved in treating PLWS.

There is an inherent challenge to creating sustained engagement with PLWS, due to the nature of schizophrenia itself. Many of the associated symptoms, including lack of insight, delusions, and social withdrawal, directly impede relationships and the desire to participate in decisions, ultimately presenting as significant therapeutic barriers. These barriers should not be seen as insurmountable, but rather factors that can be improved with PE and a focus on strong partnerships. Henceforth, the importance of individual empowerment obtained with psychosocial interventions integrated with psychopharmacological strategies should be emphasized, as this has been shown to most effectively improve patient outcomes.

This paper provides a review of the importance of therapeutic relationships and PE in the schizophrenia setting and how PLWS, clinicians and their carers need to construct a shared view relating to treatment options to increase adherence and improve outcomes. Interventions including PE can have a broad positive impact on clinical and social outcomes. We believe that future treatment can be enhanced by placing a greater emphasis on social interventions, and promoting specific training for clinicians in how to conduct difficult conversations involving delicate subject matters such as drug use.

In conclusion, we need to ensure that PLWS, carers and clinicians are properly equipped with the knowledge and skills to provide reciprocal support, increase the flow of communication, and allow PLWS to take greater ownership of their illness and its treatment.

## Author’s Note

Editorial support was provided by a medical communications agency.

## Author Contributions

All authors contributed to each stage of the development of the manuscript. Specifically, AM and WK provided input from a clinical perspective throughout. All authors contributed to the article and approved the submitted version.

## Funding

This article was made possible with funding from Janssen Pharmaceutica NV. Janssen commissioned a first draft of this article from a medical communications agency, with editing and final copy approval performed by all authors.

## Conflict of Interest

The authors declare that this study received funding from Janssen Pharmaceutica NV. The funder had the following involvement in the study: the writing of this article and the decision to submit it for publication. CM and AW are both employees of Janssen and a fee for service was provided to AM for their contribution. WK did not receive a fee for their contribution.
